# Language Proficiency Improvements After Longitudinal and Intensive Medical Spanish Courses

**DOI:** 10.7759/cureus.96010

**Published:** 2025-11-03

**Authors:** Gavin Warner, Nayantara Biswas, Marcie Naumowicz, Alejandro A Diaz, Kari E Hannibal, Jennifer Kasper, Jeffrey N Katz, Rose L Molina

**Affiliations:** 1 Department of Obstetrics and Gynecology, Beth Israel Deaconess Medical Center, Harvard Medical School, Boston, USA; 2 Program in Medical Education, Harvard Medical School, Boston, USA; 3 Department of Medicine, Brigham and Women's Hospital, Boston, USA; 4 Department of Pediatrics, Brigham and Women's Hospital, Boston, USA; 5 Department of Orthopedic Surgery, Brigham and Women's Hospital, Boston, USA

**Keywords:** assessment, curriculum development, language proficiency, medical education, spanish

## Abstract

Introduction

Few U.S. medical schools have a standardized curriculum or assessment for Medical Spanish courses, despite literature showing that language-concordant care is associated with better patient outcomes. The lack of standardized assessments of language proficiency in medical schools limits the comparison of effective instructional models.

Methods

We led an educational quality improvement study of medical students enrolled in an Intensive Medical Spanish course in Fall 2020 and Fall 2023, as well as an Advanced Medical Spanish course in Spring and Fall 2022 at one medical school. We utilized the ALTA Clinician Cultural and Linguistic Assessment (CCLA), one of the most widely used, validated language proficiency assessments for clinical communication, to evaluate students’ Spanish language proficiency before and after taking Medical Spanish courses. We analyzed pre- and post-course ALTA CCLA scores (0-100) of all students who opted in to take the assessment (16/63) using the Wilcoxon signed-rank test.

Results

Seven students took the ALTA CCLA before and after the Intensive Medical Spanish course, and nine students took the ALTA CCLA before and after the Longitudinal Medical Spanish course. Students in the intensive course had a pre-course median score of 62 (IQR 42-66.5) and a post-course median score of 80 (IQR 61.5-81). The effect size for the median score difference between the pre- and post-scores is 17.7 (95% CI: 12.0-23.0) for the intensive course and is statistically significant using the Wilcoxon signed-rank test; four of the seven students achieved “professional proficiency.” Students in the advanced course had a pre-course median score of 66 (IQR 59-74) and a post-course median score of 74 (IQR 74-82). The median score difference between the pre- and post-scores for the advanced course was 8.75 (95% CI: -4, 16), which is not a statistically significant effect size using the Wilcoxon signed rank test; four of the nine students achieved “professional proficiency.” Additionally, we found an effect size of 9.9 (95% CI: 0.3-19.4) for the difference between the intensive and advanced courses, even after controlling for the pre-course scores.

Conclusions

This educational quality improvement study shows improvements in Spanish language proficiency for students who took an Intensive Medical Spanish course.

## Introduction

Despite evidence that demonstrates improved clinical outcomes with language-concordant care [1], there is no national standard for assessing Medical Spanish proficiency. Furthermore, little is known about effective pedagogical approaches that demonstrate improvements in Medical Spanish proficiency in U.S. medical schools. Students reported that acquiring Spanish language skills in medical school was a valuable asset in their clinical practice because it enabled them to provide more equitable care through language-concordant care [2]. A national survey of 125 medical schools found that the demand for language acquisition was widespread among medical students. While 78% of medical schools had some form of Medical Spanish training, only half had a formal curriculum [3]. Medical Spanish curricula vary widely in structure, pedagogy, and duration, ranging from two-week online courses to multi-year classroom-based programs to optional standardized patient-based learning [3-6]. This variation in second language instruction may be due to limited time for elective courses because of other competing curriculum requirements and limited availability of or funding for language instructors [7].

While multiple evaluations of Medical Spanish courses have found measurable improvements in students’ Spanish language proficiency [2,4,5,8] and confidence in caring for Spanish-speaking patients [9], the studies are not consistent in which tools are used to measure improvements, limiting our ability to compare the effectiveness of different pedagogical approaches. For example, in a 2021 national survey, 43% of schools reported no post-course assessment, while 27% used standardized patient encounters, 27% written examinations, 27% telephone-based Clinician Cultural Linguistic Assessment (CCLA), 25% oral faculty-administered examinations, and 13% faculty-observed patient interviews [3]. The variation in medical school language assessments hinders alignment with national Medical Spanish competencies [10].

We utilized the ALTA Clinician Cultural and Linguistic Assessment (CCLA), a proprietary exam developed by ALTA Language Services (Atlanta, Georgia, U.S.), to evaluate medical students’ Spanish language proficiency before and after taking Medical Spanish courses. ALTA Language Services is one of the most widely published language assessments, dating back to the 2009 Kaiser Permanente study [11]. We chose the CCLA specifically because it is the most advanced option that ALTA offers and is designed for clinicians [12]. The CCLA is conducted over the phone and evaluates the individual’s ability to interact with patients using medical terminology appropriate for the clinical encounter. The CCLA rubric includes pronunciation, discourse competence, vocabulary, and cultural competency in various clinical scenarios. ALTA established a score of 80% as the threshold for “professional proficiency,” defined as qualified to communicate with patients without an interpreter, and reports the assessment has been validated [11]. While the exam has been used to assess the language proficiency of clinicians and residents who have already completed their medical training [12,13], our educational quality improvement study is among the first studies to evaluate Spanish proficiency using the ALTA CCLA among medical students who are taking two different Medical Spanish courses. This is an important distinction because medical school students are in a learning environment and have the opportunity to take medical language courses to boost their language proficiency alongside their clinical training. This study aims to evaluate differences in CCLA scores among medical students who took either an Intensive or a Longitudinal Medical Spanish course.

## Materials and methods

We led an educational quality improvement study of medical students enrolled in one of two Medical Spanish courses at Harvard Medical School: 1) Intensive Medical Spanish course in Fall 2020 and Fall 2023, or 2) Advanced Medical Spanish course in Spring and Fall 2022. Students enrolled in these courses based on their self-reported Spanish language oral proficiency as beginner, intermediate, or advanced. Additionally, instructors had 1:1 conversations with students in Spanish to verify appropriate course placement based on their perceptions of the students’ oral proficiency during the conversation. A validated placement assessment was not used to assign students to courses due to financial constraints. Additionally, there are no standardized placement exams or known cutoffs for different Medical Spanish courses. The limited operational budget for this project also limited our ability to enroll additional students.

Course structure

The Intensive Medical Spanish course was an elective designed for the beginner-intermediate level and consisted of daily online instruction (24 hours per week, Monday-Friday) for 4 weeks, taught by instructors from the International Health Central American Institute in Costa Rica. The course included grammar, clinical scenarios, simulated patient interactions, and one-on-one tutor sessions with native speakers. The syllabus included topics related to performing clinical interviews, doing physical examinations, and explaining treatment plans. Instruction also addressed common health beliefs and cultural considerations among Spanish-speaking patients, strategies for eliciting the chief complaint, and the use of both colloquial and medical terminology to support effective clinical communication. Due to curricular constraints, only third- and fourth-year medical students were enrolled in the course because they were the only ones who could fit a month-long elective during their course schedule.

The longitudinal Advanced Medical Spanish course was an elective consisting of 1.5 hours of weekly instruction for 13 weeks, with most sessions held via the Zoom remote platform (Zoom Communications, San Jose, California, U.S.) and two sessions held in person due to the COVID-19 pandemic. The course was taught by a native Spanish-speaking physician. The course focused on oral skills for clinical interactions, and students practiced speaking with native speakers. The syllabus included topics related to obtaining a history (e.g., chief concern, history of present illness, past medical/surgical history, and family history). Instruction also incorporated culturally specific scenarios, such as diet counseling and discussions of cultural values like familismo (the centrality of family) and respeto (respect for authority and hierarchy) in Hispanic healthcare contexts. Due to curricular constraints, mostly first-year medical students enrolled in the course because it was held in the evenings after their other required courses. 

Inclusion and exclusion criteria 

Course directors of the Intensive Medical Spanish course in 2020 and 2023 and the Advanced Medical Spanish course in Spring 2022 invited all enrolled students via email to complete an optional ALTA CCLA by phone on their own schedule, both at the beginning and at the end of the course. In total, 55 students from those three courses were invited to participate. All students who volunteered to take the ALTA exam and completed both the pre- and post-test were included in the study. Due to the limited budget for the exams, we were unable to require all students to take the assessment before and after the intensive courses in 2020 and 2023. In Fall 2022, the CCLA was required for all eight students enrolled in the Advanced Medical Spanish course. Seven were included in the study due to one student not completing the post-course CCLA. We did not collect demographic information from students because this was an educational quality improvement study; we wanted to minimize the participation burden on students during the COVID-19 pandemic, which limits the generalizability of our findings beyond the specific educational context at our institution. The Harvard Medical School Educational Scholarly Review Committee determined this study was for educational quality improvement, so it did not meet the criteria for human subject research under the purview of the IRB.

Data analysis 

Because of the non-normal data distribution, we analyzed students’ pre- and post-course ALTA CCLA score difference (0-100) using the Wilcoxon signed-rank test and measured each student’s improvement in Medical Spanish proficiency over the course period with statistical significance set at p<0.05. Because of the small heterogeneous sample based on course year, students’ prior proficiency, and differing instructional design, we only tested the statistical significance of student scores within the Intensive and Advanced courses and not across courses. We report differences in CCLA scores between the two course formats descriptively, comparing the median improvement, percent change from the baseline scores, and the number of students that reached the “professional proficiency” threshold. We report each individual student’s paired pre-course and post-course scores, allowing visualization of students who approached the “professional proficiency” threshold of 80. We also performed a post-hoc power analysis and calculated an effect size to capture the differential impact between the courses using simple linear regression. 

## Results

Of the 23 students enrolled in the Intensive Spanish Fall 2020 course, 5 completed both the pre-course and the post-course ALTA CCLA. Of the 28 students enrolled in the same course in Fall 2023, 2 completed both exams. In total, only 14% of eligible students completed both pre- and post-course assessments. There were five female and two male students from the Intensive Medical Spanish. Six were fourth-year medical students, and one was a third-year medical student. All students in this course had beginner-intermediate proficiency in Spanish at baseline.

Of the eight students enrolled in the Advanced Spanish Fall 2022, seven completed both the pre-course and post-course ALTA CCLA. In Fall 2023, four students enrolled, of whom two completed both exams. In total, 75% of eligible students completed both pre- and post-course assessments. There were seven female and two male students who took the pre- and post-course ALTA CCLA from the Advanced Spanish course. Five were first-year medical students, one was a third-year medical student, and three were public health students. All students in this course had prior Spanish experience with an advanced-level baseline.

**Figure 1:**
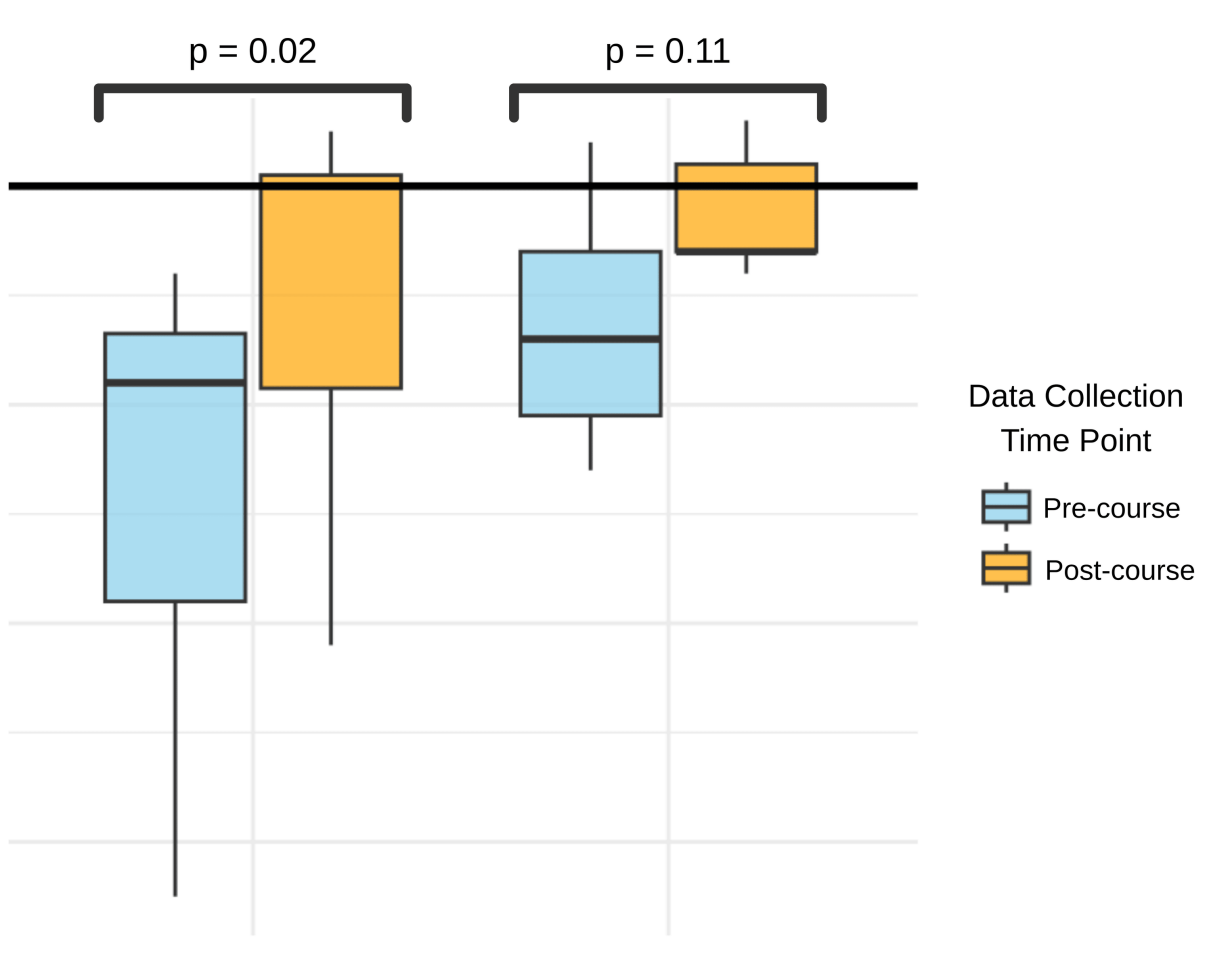
ALTA Clinician Cultural and Linguistic Assessment (CCLA) pre- and post-course scores in Intensive and Advanced Medical Spanish courses Possible score of 0-100, with 80 as the threshold for “professional proficiency” for direct communication with patients.

**Figure 2:**
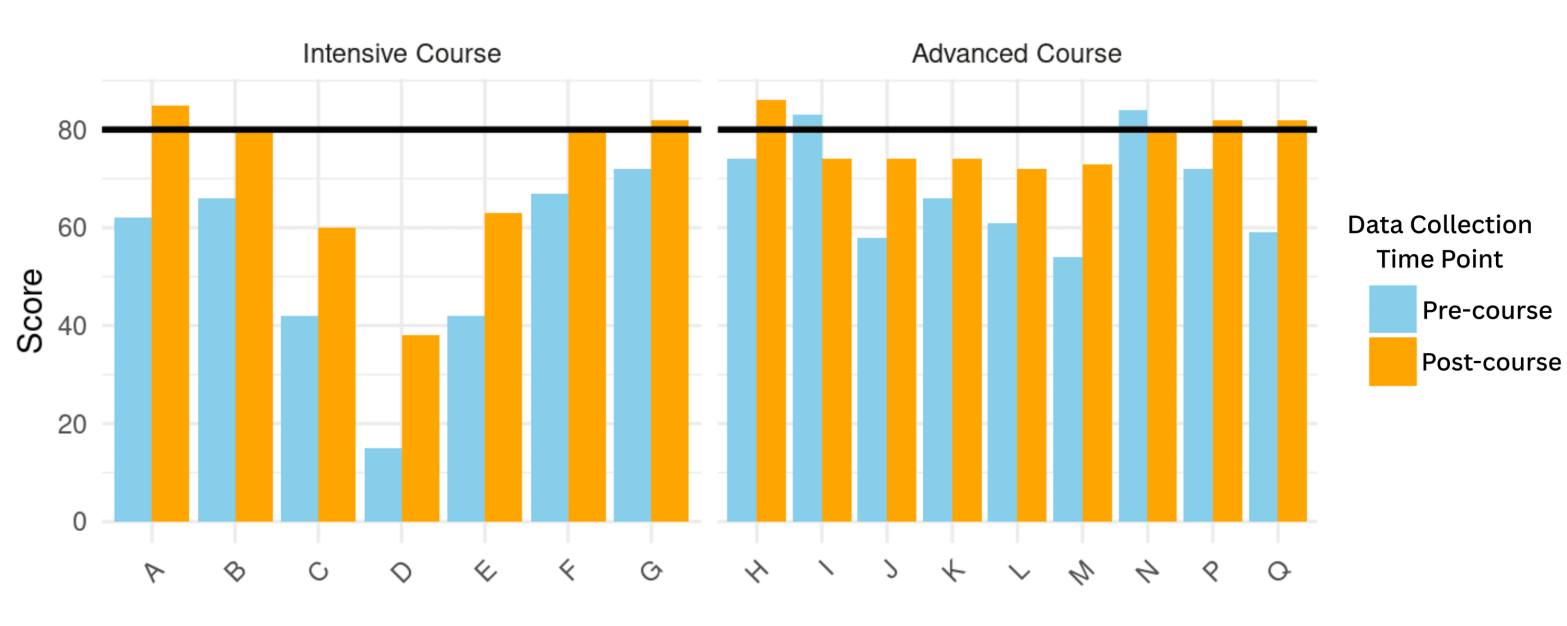
ALTA Clinician Cultural and Linguistic Assessment (CCLA) paired pre- and post-scores in Intensive and Advanced Medical Spanish courses Possible score of 0-100, with 80 as the threshold for “professional proficiency” for direct communication with patients. A-Q indicate the individual student’s paired scores.

Students in the intensive course had a pre-course median score of 62 (IQR 42-66.5) and a post-course median score of 80 (IQR 61.5-81) upon completing the course (Figure 1). The difference in the median score was 17.7 and statistically significant with a p-value of 0.02 (95% CI: 12.0 - 23.0), using the Wilcoxon signed-rank test. Notably, four of the seven students achieved “professional proficiency” by passing the threshold of 80 after the month-long course (Figure 2).

Students in the advanced course had a pre-course median score of 66 (IQR 59-74) and a post-course median score of 74 (IQR 74-82) (Figure 1). The median score difference was 8.8, using the Wilcoxon signed-rank test, but this effect size did not reach statistical significance (p=0.11; 95% CI: -4.0 - 16.0). Four of the nine students achieved “professional proficiency” after taking the course (Figure 2). Students who approached “professional proficiency” are shown in Figure 2.

Students in the intensive course showed significantly greater improvement in pre- to post-course scores than those in the advanced course. We found an effect size of β coefficient = 9.86 (95% CI: (0.33, 19.39), p = 0.044), indicating that students in the intensive course improved their scores by nearly 10 points more than those in the advanced course, after adjusting for baseline (i.e., pre-course) performance. This suggests that course format might play a role in clinical communication proficiency. However, a post hoc power calculation revealed that the study had only 52.7% power to detect this 9.86-point difference, suggesting limited precision and an increased risk of Type II errors. Thus, while statistically significant, the finding should be interpreted cautiously, and future studies with larger sample sizes are warranted to confirm this effect.

## Discussion

We led an educational quality improvement study of Medical Spanish oral proficiency among medical students enrolled in a beginner-intermediate Intensive Medical Spanish course and a longitudinal Advanced Medical Spanish course. We analyzed students’ Medical Spanish proficiency improvement using the same standardized assessment and found a statistically significant 29% improvement in pre- to post-course scores among students in the Intensive course. We found a 17% improvement in scores for students in the Advanced course, but this was not statistically significant due to limited power. While students in the longitudinal course began with higher baseline Spanish proficiency, the results of this sample of 16 participants suggest the intensive course supported beginner-to-intermediate-level students in achieving larger gains in oral Spanish skills. In fact, two students from the Advanced course had achieved “professional proficiency” in the pre-test. At the end of the course, the Intensive group matched the Advanced group in the number of students who achieved “professional proficiency” in the post-course assessment, with four students being qualified to speak with patients in Spanish. However, one student from the Advanced group who had previously reached this threshold did not maintain it in the post-test, which may be due to test error or performance variability. A larger sample size is needed to verify proficiency gains across courses. Even though the course structure and formats were different, precluding direct comparison, the course differences reflect curricular constraints present at many medical schools.

The results of our study suggest the potential of concentrated, high-exposure language instruction in improving language proficiency as measured by the ALTA CCLA. Such findings are consistent with a study in which all students who completed a one to two-month-long clinical elective abroad reported improved language skills [14]. Although the intensive course and the elective abroad differ substantially in terms of immersion learning environment, there is little published about intensive language training during medical school in the U.S. [15]. Longitudinal Spanish courses are also useful in facilitating longer engagement and retention of skills over time and may be more feasible based on scheduling constraints. One study surveyed students one year after they took a 10-week-long Medical Spanish elective and found 73.0% of students were still comfortable in performing a simple, problem-focused patient interview in Spanish [2]. Future studies could explore how well students are able to retain their Spanish-speaking ability after an intensive course. While our study did not find statistically significant results in the advanced group, this could be due to not having adequate power to detect a difference.

In the 2015 national survey of Medical Spanish curricula, the most common barrier to schools’ implementation of Medical Spanish courses was a lack of time in students’ schedules [7]. Our study includes two types of Medical Spanish courses with differing lengths of time, which may be helpful for schools to consider based on other curricular scheduling constraints. A month-long intensive elective course may help students gain Spanish-speaking skills while minimizing the additional burden of the competing demands of medical school. 

Additionally, our study underscores that having advanced Spanish-language proficiency does not automatically qualify students to safely communicate with patients in that language. More than half of the students who identified as having advanced language proficiency did not meet the ALTA CCLA threshold for “professional proficiency.” This may point to their lack of context-specific medical Spanish language skills rather than their general Spanish proficiency. A recent study has shown that being a heritage Spanish speaker does not guarantee fluency to communicate safely with patients in clinical settings due to unfamiliar complex medical terms [16]. This underscores the importance of having language assessment standards tailored to clinicians to verify the appropriate skills needed for safe patient communication.

Ensuring clinicians’ oral language proficiency to communicate with patients in a non-English language is critical for avoiding safety errors and ensuring quality of care [17,18]. In an analysis of 35 malpractice cases related to the failure to provide appropriate language services, 32 of the cases occurred when the health care providers did not include qualified interpreters [19]. This highlights the risk of relying on individuals without verified oral proficiency and the need to provide training and assessment to medical school students who speak languages other than English. 

While this study is one of the first to report students’ medical Spanish language proficiency improvement in different courses based on an objective assessment, there are other validated tools, such as the Physician Oral Language Observation Matrix (POLOM), which have been used to evaluate Spanish proficiency in a clinical education setting [20]. Future studies could compare assessments, such as the ALTA CCLA and the POLOM, in the context of language curricula in medical schools. Because there is no national standard for clinical Spanish certification, the ALTA CCLA and the POLOM could be tools to provide some standardization until the development of a standardized national certification exam in Medical Spanish [10].

Our study had several limitations. Our sample size was small, limiting precision, and included only one institution, limiting generalizability. The low response rate (25%) raises concerns about selection bias and potential overestimation, as students who self-selected to take the assessment may have been more confident in their Spanish abilities than those who did not. Some reasons for not participating in the study could be the optional nature of the assessment due to budget limitations and competing priorities in medical students’ schedules, especially during the COVID-19 pandemic. Another limitation is the subjective course placement process that could have introduced inaccuracies, as students with higher proficiency could have been misplaced in the intensive course due to scheduling availability. Differences in clinical experience between first-year, upper-year, and public health students also may have influenced performance on the CCLA, independent of Spanish language acquisition, making first-year medical students and public health students appear to have lower CCLA scores based on their limited clinical training. We were unable to collect students’ self-reported demographics, which limits our ability to control for confounding factors like prior language exposure and time spent in Spanish-speaking environments. Differing course content and pedagogy preclude direct comparison between the intensive and longitudinal courses. Lastly, using ALTA CCLA may be a limitation in the absence of a gold standard objective language proficiency assessment for medical students.

## Conclusions

Our findings in this educational quality improvement study suggest that the Intensive Medical Spanish course may help medical students with intermediate-level skills achieve meaningful improvements in language proficiency. Additionally, the Longitudinal Advanced Medical Spanish course may also improve students’ language proficiency, but our study was underpowered to detect meaningful differences in students’ pre- and post-course ALTA CCLA scores. As medical schools seek effective ways to prepare students to care for Spanish-speaking patients, intensive language courses may offer a valuable addition to the curriculum. Future studies with larger sample sizes are needed to provide more generalizable results to other educational settings.
